# Epidemiologic Characteristics of and Prognostic Factors for COVID-19 Among Hospitalized Patients: Updated Implications From Hubei Province, China

**DOI:** 10.3389/fpubh.2021.726491

**Published:** 2021-10-27

**Authors:** Xiang Liu, Linzhi Zhu, Tingjuan Lu, Xibang Liu, Demin Jiao, Xiali Tang, Jun Chen, Yu Chen, Wenya Yu, Qingyong Chen

**Affiliations:** ^1^Department of Respiratory Disease, The 903rd Hospital of the People's Liberation Army, Hangzhou, China; ^2^School of Public Health, Shanghai Jiao Tong University School of Medicine, Shanghai, China; ^3^The First Affiliated Hospital of Wenzhou Medical University, Wenzhou, China; ^4^The Optics Valley Branch of the Maternal and Child Hospital of Hubei Province, Wuhan, China

**Keywords:** COVID-19, indicator, pandemic, prognosis, prognostic factor

## Abstract

**Introduction:** The roles of some indicators in the prognosis of patients with coronavirus disease-19 (COVID-19) remain unclear and controversial. This study aimed to explore the epidemiologic characteristics of and prognostic factors for COVID-19 to provide updated recommendations for its prevention, diagnosis, and treatment.

**Methods:** For this retrospective study, demographic, epidemiologic, and clinical data were extracted from the medical records of patients admitted to the Maternal and Child Hospital of Hubei Province (Optical Valley) with COVID-19 between February 19, 2020, and March 19, 2020. The primary outcome was the prognosis that was determined at discharge as mentioned in the medical records. Descriptive statistics, univariate analyses, and stepwise logistic regression analysis were used for data analysis.

**Results:** Of the 1,765 patients included, 93.1% were cured and the mortality was 1.8%. Univariate analyses identified 63 factors significantly associated with COVID-19 prognosis. Logistic regression analysis revealed that a poorer prognosis was associated with undergoing resuscitation, complex disease manifestations, consultation with outside specialists, elevated basophil or lymphocyte counts, an albumin (ALB)/globulin (A/G) ratio > 2.4, and elevated levels of serum aspartate aminotransferase (AST) or creatinine. Patients had a better prognosis if the following conditions were met: dry cough reported as an initial symptom, fatigue as a clinical manifestation, and a diagnosis based on laboratory testing.

**Conclusion:** To prevent clinical deterioration, clinicians should provide special care to patients who underwent resuscitation, with a critical disease, or requiring consultation with outside specialists. Extra attention should be paid to patients with high basophil or lymphocyte counts, a high A/G ratio, and elevated AST or creatinine levels.

## Introduction

In early December 2019, several pneumonia cases of unknown origin emerged in Wuhan, China, later attributed to a novel coronavirus (1, 2). On February 11, 2020, the WHO officially named the disease coronavirus disease-19 (COVID-19) (3). Since then, COVID-19 has spread globally, and on March 11, 2020, the WHO declared COVID-19 a pandemic (3). By July 30, 2021, there had been 196,553,009 confirmed cases of COVID-19, of which 4,200,412 deaths were reported to the WHO, of which 102,553 confirmed cases and 5,635 deaths were from China (4). Although great progress has been achieved in epidemiological experience, clinical management, and vaccination, the pandemic continues to progress globally.

Because China was the first country to report the outbreak of COVID-19, Chinese clinicians and researchers have continued to explore and update the assessment of epidemic characteristics and COVID-19 prognostic factors, providing evidence for its prevention, diagnosis, and treatment. The prognostic factors include demographic characteristics (5), underlying diseases (6), laboratory parameters (7, 8), clinical manifestations (9, 10), radiological features (11), and treatment (12). In addition, vaccination becomes a positive prognostic factor recently, with more than 10 vaccines of different strategies being reported to reduce the severity of the disease (13–17). Similar findings regarding the aforementioned prognostic factors have been reported from various countries, namely, France (18), Saudi Arabia (19), Spain (20), Italy (21), and others (22).

Despite overall consistency in findings between different studies, data on several prognostic factors, such as sex (23, 24), clinical manifestations (25), and underlying diseases (25, 26), remain inconsistent or variable. For example, the African COVID-19 Critical Care Outcomes Study revealed that sex was not independently associated with outcome (24), while a study in Detroit revealed that male sex was significantly associated with mortality (23). Though having chronic diseases has been reported to be a risk factor for worse prognosis in some studies, other studies have found that they had no influence on prognosis (25–28). In addition, many studies had a small sample size, which may cause biases. Some prognostic factors, such as some laboratory test results, radiological features, and treatments, have not been explored owing to limited data availability.

Because there remains much about COVID-19 that is unknown or poorly understood and the disease continues to be prevalent and evolve as new variants emerge, it is important to understand demographic, epidemiologic characteristics, clinical features, laboratory test results, radiological features, and treatment, especially the controversial indicators or those not explored in previous studies. Therefore, to gain insight into these inconsistencies and expand the understanding of additional indicators, this study aimed to explore the epidemiologic characteristics and factors influencing COVID-19 prognosis with a relatively large sample size. The findings of the potential prognostic indicators from this study will provide an updated assessment of their implications for the prevention, diagnosis, and treatment of COVID-19.

## Methods

### Study Design and Participants

The study was a retrospective review of patients with COVID-19 hospitalized at the Optics Valley Branch of the Maternal and Child Hospital of Hubei Province (MCH-Optical Valley). During the first wave of the COVID-19 epidemic in Wuhan, MCH-Optical Valley was a designated hospital for the treatment of patients with COVID-19, starting on February 13, 2020. Eligible patients for this study included all inpatients admitted to MCH-Optical Valley with confirmed or suspected COVID-19 between February 19, 2020, and March 19, 2020. The prognosis was defined as the primary outcome at discharge as mentioned in medical records. All patients were discharged between February 19, 2020, and April 5, 2020.

### Data Collection

Medical records of patients at MCH-Optical Valley were obtained from the electronic medical record system. The inclusion criteria were (1) hospitalization owing to confirmed or suspected COVID-19; (2) hospitalization at MCH-Optical Valley between February 19, 2020, and March 19, 2020; and (3) complete medical records covering the seven dimensions mentioned below. The exclusion criteria were (1) outpatient consultation; (2) hospitalized during other time periods; and (3) in complete medical records. Finally, a total of 1,765 patients with detailed records were included in this study. Data in this medical record system could be divided into seven dimensions: (1) demographic characteristics, (2) epidemiological exposure history, (3) admission characteristics, (4) hospitalization and treatment characteristics, (5) imaging features, (6) laboratory findings, and (7) diagnosis and prognosis.

Demographic characteristics included sex, marital status, occupation, and age. Epidemiological exposure history referred to exposure history (contact with someone with confirmed or suspected COVID-19 during the 2 weeks preceding the onset of illness), the relationship between patients and people with COVID-19 to whom they were exposed, source of exposure, time period of exposure, and possible location of exposure. Admission characteristics included admission route, illness condition on admission, critical degree on admission, and vital signs on admission (body temperature, pulse rate, respiratory rate, systolic blood pressure [SBP], and diastolic blood pressure [DBP]). Hospitalization and treatment characteristics included whether patients underwent surgery, underwent resuscitation (life-threatening patients needing rescue), developed critical illness, required consultation, had complex cases, reported initial symptoms, current symptoms during hospitalization, clinical manifestations, previous medical history, self-reported underlying diseases, smoking habits, treatment, and length of stay. Imaging features on CT or chest radiography included ground-glass opacity (GGO) characteristics, location, and other observations. Laboratory findings were classified into two categories (according to the sample), with a total of 44 indicators. The first category comprised the following indicators tested using whole blood: C-reactive protein (CRP), white blood cell (WBC) count, percentage of lymphocytes, percentage of eosinophils, neutrophil count, monocyte count, basophil count, hemoglobin level, mean corpuscular volume, mean corpuscular hemoglobin concentration (MCHC), platelet (PLT) count, percentage of neutrophils, percentage of monocytes, percentage of basophils, lymphocyte count, eosinophil count, red blood cell count, packed cell volume, mean corpuscular hemoglobin, red blood cell distribution width (RDW), and mean platelet volume. The second category comprised the following indicators tested using serum: blood glucose, albumin (ALB), ALB/globulin (A/G) ratio, direct bilirubin (D-BiL), alanine aminotransferase, alkaline phosphatase (ALP), total bile acid, sodium, calcium, serum magnesium, urea nitrogen (UN), uric acid, total protein (TP), globulin, total bilirubin, indirect bilirubin (I-BiL), aspartate aminotransferase (AST), γ-glutamyl transpeptidase, kalium, chlorine, phosphorus, total carbon dioxide, and creatinine. Diagnosis and prognosis considered outpatient and emergency diagnosis, admission diagnosis, discharge diagnosis, a subtype of COVID-19 at discharge (mild, normal, severe, critical, suspected, and clinical diagnosis subtypes), consistency between discharge and outpatient diagnoses, consistency between admission and discharge diagnoses, consistency between preoperative and postoperative diagnoses, consistency between clinical and pathological diagnoses, consistency between radiological and pathological diagnoses, the highest evidence of diagnosis (clinical diagnosis; radiography, CT, ultrasound, and endoscopy; biochemical and immunological test results; and cytological blood smear test), and the prognosis at discharge (dead, unhealed, improved, and cured).

### Data Analysis

Statistical analyses, such as descriptive statistics, univariate analyses, and multivariate analysis, were conducted using SAS 8.2 (SAS Institute Inc., Cary, NC, USA) and SPSS 18.0 (SPSS Inc., Chicago, IL, USA). First, descriptive statistics were used to describe the frequency and percentage of categorical variables. Continuous variables are presented as the median and interquartile ranges because all continuous variables had a skewed distribution. Second, univariate analyses were conducted to determine the association between prognosis and each variable. Specifically, the Wilcoxon rank test was used to test the relationship between prognosis and dichotomous variables (e.g., sex). The Kruskal-Wallis H test was used to test the relationship between prognosis and multivariate variables (e.g., marital status). The Spearman rank correlation test was used to test the relationship between prognosis and continuous variables (e.g., age). Third, because the prognosis (the dependent variable) was an ordinal multi-categorical variable, stepwise logistic regression analysis was used to explore the factors influencing COVID-19 prognosis. Factors having a significant association with prognosis in the univariate analysis were included in the logistic model, with the significance level for entry set at 0.10 and the significance level for selection set at 0.15. To explain the results more explicitly, continuous variables were transformed into categorical variables according to their meanings and clinical reference ranges in the logistic regression analysis. All tests were two-sided, and *P* < 0.05 were considered statistically significant.

### Ethical Approval and Patient Consent

This study was approved by the 903rd Hospital of PLA ethics committee (approval reference number: 20210224/02/01/002). The requirement for informed consent was waived by the ethics committee because of the retrospective nature of the study and the urgent nature of the pandemic.

## Results

### Demographic and Epidemic Characteristics of Patients With COVID-19

As shown in Table 1, 58.4% of the 1,765 patients with COVID-19 were women, 89.6% were married, and 43.3% were retired. The average age was 58.7 ± 15.1 years (Supplementary Table 1).

**Table 1:** Basic characteristics of patients with COVID-19.

**Characteristics**	*N* ^*^	%^*^
**Total**	**1,765**	**1.0**
**Sex**		
Male	734	41.6
Female	1,031	58.4
**Marital status**		
Unmarried	67	3.8
Married	1,582	89.6
Widowed	90	5.1
Divorced	26	1.5
**Occupation**		
Worker	86	4.9
Farmer	75	4.2
Office worker	179	10.1
Civil servant	44	2.5
Professional and technological worker	48	2.7
Self-employed person	44	2.5
Freelancer	115	6.5
Student	16	0.9
Retiree	764	43.3
Unemployed	197	11.2
Others	197	11.2
**Exposure history (contact with someone with confirmed or suspected COVID-19 during the 2 weeks preceding the onset of illness)**		
No	998	56.5
Yes	477	27.0
Unknown	290	16.4
**Relationship between patients and people with COVID-19 to whom they were exposed**		
Family members	330	18.7
Colleagues	36	2.0
Social interaction	50	2.8
Shared transportation	12	0.7
Others	1,337	75.8
**Source of exposure**		
Eating together	125	7.1
Staying in the same room	218	12.4
Living in the same ward	17	1.0
Sharing utensils with patients	5	0.3
Contacting with patient secretions	4	0.2
Treatment and care	828	46.9
Visiting patient	5	0.3
Others	563	31.9
**Time period of exposure**		
Prolonged	345	19.5
Brief	100	5.7
Uncertain	1,320	74.8
**Possible location of exposure**		
Home	307	17.4
Workplace	40	2.3
Dormitories	13	0.7
Hospitals	31	1.8
Indoor public places	40	2.3
Others	945	53.5
**Admission route**		
Emergency department	23	1.3
Outpatient department	47	2.7
Referral from other hospitals	119	6.7
Others	1,576	89.3
**Illness condition on admission**		
Without symptoms	10	0.6
Unknown	3	0.2
Clinically uncertain	20	1.1
With symptoms	1,732	98.1
**Critical degree on admission**		
Dangerous	72	4.1
Emergent	66	3.7
Moderate	1,627	92.2
**Undergoing surgery during hospitalization**		
No	1,757	99.5
Yes	8	0.5
**Undergoing resuscitation during hospitalization**		
No	1,707	96.7
Yes	58	3.3
**Developing critical illness during hospitalization**		
No	1,559	88.3
Yes	206	11.7
**Requiring consultation during hospitalization**		
No	1,203	68.2
In-hospital consultation	93	5.3
Consultation with outside specialists	469	26.6
**Being complex cases during hospitalization**		
No	1,731	98.1
Yes	34	1.9
**Reported initial symptoms**		
Fever	959	54.3
Cough	787	44.6
Expectoration	112	6.3
Dry cough	159	9.0
Stuffy nose and/or runny nose	18	1.0
Pant	162	9.2
Shortness of breath	126	7.1
Fatigue	537	30.4
Chest distress and/or chest pain	237	13.4
Dizziness and/or headache	28	1.6
Abdominal pain and/or diarrhea and/or bloating	38	2.2
Sore throat	58	3.3
Dyspnea	31	1.8
Hemoptysis	2	0.1
Palpitation	19	1.1
Muscle pain	45	2.5
Chest and/or back pain	7	0.4
Nausea and/or vomit	21	1.2
Poor appetite	49	2.8
Chill	25	1.4
Disturbance of consciousness and apathy	10	0.6
Viral pneumonia	2	0.1
Tuberculosis	1	0.1
Unknown	18	1.0
**Current symptoms during hospitalization**		
Fever	1,059	60.0
Cough	1,084	61.4
Chest distress	390	22.1
Dyspnea	77	4.4
**Clinical manifestations**		
Fever	732	41.5
Cough	800	45.3
Catarrh of the upper respiratory tract	196	11.1
Chest distress	476	27.0
Dyspnea	176	10.0
Fatigue	658	37.3
Diarrhea	74	4.2
**Previous medical history**		
Without	1,263	71.6
With	502	28.4
**Self-reported underlying diseases**		
Hypertension	528	29.9
Diabetes	226	12.8
Coronary heart disease	100	5.7
Chronic bronchitis	41	2.3
COPD	9	0.5
Hyperlipoidemia	23	1.3
Asthma	14	0.8
Atrial fibrillation	12	0.7
Bronchiectasis	8	0.5
Alzheimer's disease	15	0.8
Parkinson's disease	6	0.3
**Smoking habits**		
No	1,387	78.6
Yes	74	4.2
Unknown	304	17.2
**Treatment**		
Oxygen therapy measures	677	38.4
Antiviral therapy	1,255	71.1
Mechanical Ventilation	30	1.7
ECMO	4	0.2
Circulatory support	2	0.1
Renal failure and renal replacement therapy	2	0.1
Blood purification therapy	2	0.1
Immunotherapy with tocilizumab	20	1.1
Severe or critical child cases	9	0.5
Pregnancy with severe or critical illness	1	0.1
**Ground-glass opacity tested by CT or chest radiography**		
No	231	13.1
Yes	1,534	86.9
**Location of ground-glass opacity**		
Only right lung	135	7.6
Only left lung	93	5.3
Multiple points of both lungs	1,226	69.5
Right and upper left lung	31	1.8
Right and lower left lung	32	1.8
Left and upper right lung	1	0.1
Left and middle right lung	10	0.6
Left and lower right lung	8	0.5
Unknown	229	13
**Other observations obtained from imaging**		
Bronchial vascular bundle thickening	493	27.9
Swollen lymph node	174	9.9
Pleural effusion	91	5.2
**Outpatient and emergency diagnosis**		
Confirmed COVID-19 case	1,331	75.4
Clinically diagnosed COVID-19 case	266	15.1
Suspected COVID-19 case	140	7.9
Infected by novel coronavirus	14	0.8
Viral pneumonia	13	0.7
Hypertension	1	0.1
**Admission diagnosis**		
Confirmed COVID-19 case	1,329	75.3
Clinically diagnosed COVID-19 case	289	16.4
Suspected COVID-19 case	125	7.1
Infected by novel coronavirus	14	0.8
Viral pneumonia	5	0.3
Hypertension	2	0.1
Chronic bronchitis	1	0.1
**Discharge diagnosis**		
Confirmed COVID-19 case	1,536	87.0
Clinically diagnosed COVID-19 case	200	11.3
Suspected COVID-19 case	14	0.8
Infected by novel coronavirus	14	0.8
Pneumonia	1	0.1
**Subtype of COVID-19 at discharge**		
Mild	25	1.4
Normal	928	52.6
Severe	60	3.4
Critical	26	1.5
Suspected	7	0.4
Clinical diagnosis	194	11.0
Unknown	525	29.7
**Consistency between discharge and outpatient diagnoses**		
Consistent	1,730	98.0
Uncertain	2	0.1
Unknown	33	1.9
**Consistency between admission and discharge diagnoses**		
Consistent	1,755	99.4
Uncertain	2	0.1
Unknown	8	0.5
**Consistency between preoperative and postoperative diagnoses**		
Inconsistent	14	0.8
Consistent	125	7.1
Uncertain	2	0.1
Unknown	1,624	92.0
**Consistency between clinical and pathological diagnoses**		
Inconsistent	14	0.8
Consistent	13	0.7
Uncertain	1	0.1
Unknown	1,737	98.4
**Consistency between radiological and pathological diagnoses**		
Inconsistent	14	0.8
Consistent	23	1.3
Uncertain	2	0.1
Unknown	1,726	97.8
**The highest evidence of diagnosis**		
Clinical diagnosis	414	23.5
Radiography, CT, ultrasound, endoscopy	107	6.1
Biochemical and immunological test results	928	52.6
Cytological blood smear test	21	1.2
Unknown	295	16.7
**Prognosis at discharge**		
Dead	32	1.8
Unhealed	4	0.2
Improved	85	4.8
Cured	1,644	93.1

* *The frequency and percentage of these categorical variables were calculated by the descriptive statistics. COVID-19, coronavirus disease-19*.

Of the total number of patients, 27.0% had an exposure history of contact with someone with suspected or confirmed COVID-19 during the 2 weeks preceding the onset of their illness. Most contacts (75.8%) were with people other than family members, colleagues, and people with social interactions and shared transportation. The most common source of exposure was through treatment and care. Of the patients, 74.8% had an uncertain time period of exposure and 53.5% were exposed in locations other than their home, workplace, dormitories, hospitals, or indoor public places (Table 1).

Most patients were admitted to the hospital through other routes (rather than through the emergency department, outpatient department, or referral from other hospitals); 98.1% had symptoms at the time of admission, and 92.2% had moderately severe disease on admission (Table 1). The vital signs on admission, such as body temperature, pulse rate, respiratory rate, SBP, and DBP, are shown in Supplementary Table 1.

The average length of stay was 13.3 ± 6.1 days (Supplementary Table 1). During hospitalization, most did not undergo surgery (99.5%) or resuscitation (96.7%), did not develop critical illness (88.3%) or require consultation with outside specialists (68.2%), and did not have complex cases. The top three initial symptoms reported were fever, cough, and fatigue. Over 60% of the patients experienced cough (61.4%) and fever (60.0%) during hospitalization. The top three clinical manifestations were consistent with the reported initial symptoms. Most patients had no previous medical history of note. The top three self-reported underlying diseases were hypertension, diabetes, and coronary heart disease. Of the patients, 78.6% were non-smokers. Most received antiviral therapy during hospitalization. Chest CT or radiography showed that most had GGO, observed at multiple points in both lungs. In addition, 27.9% of the patients had bronchial vascular bundle thickening (Table 1). The results of laboratory tests that include the 44 indicators are shown in Supplementary Table 1.

Most patients were diagnosed with confirmed COVID-19 at the time of outpatient or emergency department consultation, admission, and discharge. Over 50% of the patients were diagnosed with a normal subtype of COVID-19 at discharge. For most patients, the discharge and outpatient diagnoses and the admission and discharge diagnoses were consistent. However, the preoperative and postoperative diagnoses, clinical and pathological diagnoses, and radiological and pathological diagnoses could not be compared because of insufficient data. In terms of the diagnoses mentioned above, 52.6% of patients were diagnosed based on the highest level of evidence of biochemical and immunological tests, indicating that most (93.1%) patients were cured at discharge and the case fatality rate was 1.8% (Table 1).

### Univariate Analysis of Factors Influencing COVID-19 Prognosis

Univariate analyses (Supplementary Tables 1, 2) suggested the following 63 factors significant to the prognosis: marital status; occupation; age; exposure history (contact with someone with confirmed or suspected COVID-19 during the 2 weeks preceding the onset of illness); source of exposure; time period of exposure; critical degree on admission; undergoing surgery; undergoing resuscitation; critical disease; need for consulting outside specialists; complex disease manifestations; reported initial symptom of dry cough, fatigue, dyspnea, disturbance of consciousness and apathy, and unknown; current symptom of dyspnea; clinical manifestation of fever, cough, and fatigue; medical history; self-reported underlying hypertension, diabetes, atrial fibrillation, and Alzheimer's disease; smoking habits; treatment method of oxygen therapy measures, mechanical ventilation, and immunotherapy with tocilizumab; imaging feature of pleural effusion; lymphocyte count; percentage of lymphocytes; percentage of eosinophils; neutrophil count; basophil count; MCHC; PLT count; percentage of neutrophils; percentage of monocytes; percentage of basophils; lymphocyte count; eosinophil count; RDW; blood glucose; ALB; A/G ratio; D-BiL; ALP; UN; TP; AST; chlorine; creatinine; CRP; outpatient and emergency diagnosis; admission diagnosis; discharge diagnosis; subtype of COVID-19 at discharge; consistency between discharge and outpatient diagnoses; consistency between admission and discharge diagnoses; consistency between preoperative and postoperative diagnoses; and the highest level of evidence of diagnosis.

### Logistic Regression Analysis of COVID-19 Prognosis

Logistic regression analysis (Table 2) revealed that the prognosis of patients with COVID-19 was influenced by the following factors: undergoing resuscitation, developing a critical illness, requiring outside specialist consultation, having dry cough as an initial symptom, the clinical manifestation of fatigue, the highest level of evidence of diagnosis, basophil count, lymphocyte count, A/G ratio, and AST and creatinine levels.

**Table 2:** Logistic regression analysis of the influencing factors of the prognosis of patients with COVID-19.

**Characteristic**		**Estimate^a^**	**Wald Chi-Square^a^**	***P* ^a^**	**OR^a^**	**95% Wald confidence limits** ^a^	
						**Lower**	**Upper**
Critical degree on admission	Dangerous	Ref					
	Emergent	−0.727	2.458	0.117	0.483	0.195	1.200
	Moderate	0.410	0.982	0.322	1.507	0.670	3.393
Undergoing resuscitation during hospitalization	No	Ref					
	Yes	−2.700	48.307	<0.0001^*^	0.067	0.031	0.144
Developing critical illness during hospitalization	No	Ref					
	Yes	−0.856	6.441	0.011^*^	0.425	0.219	0.823
Requiring consultation during hospitalization	No	Ref					
	In-hospital consultation	−0.650	3.146	0.076	0.522	0.255	1.071
	Consultation with outside specialists	−2.559	14.310	0.000^*^	0.077	0.021	0.291
Having a dry cough as an initial symptom	No	Ref					
	Yes	1.820	5.510	0.019^*^	6.170	1.350	28.197
Having an unknown initial symptom	No	Ref					
	Yes	−1.210	3.541	0.060	0.298	0.085	1.052
Clinical manifestation of fatigue	No	Ref					
	Yes	0.673	7.234	0.007^*^	1.960	1.200	3.200
The highest evidence of diagnosis	Clinical diagnosis	Ref					
	X-ray, CT, ultrasound, endoscopy	−0.128	0.089	0.766	0.880	0.378	2.046
	Biochemical and immunological test	0.873	10.990	0.001^*^	2.395	1.429	4.014
	Cytological blood smear	−1.023	2.490	0.115	0.359	0.101	1.281
	Unknown	0.117	0.135	0.713	1.124	0.604	2.091
Basophil count (×10^9^/L)^b^	0–0.06	Ref					
	>0.06	−0.927	5.797	0.016^*^	0.396	0.186	0.842
Lymphocyte count (×10^9^/L)^b^	1.1–3.2	Ref					
	<1.1	−0.098	0.123	0.726	0.907	0.525	1.566
	>3.2	−1.554	6.731	0.010^*^	0.211	0.065	0.684
Albumin (ALB) / globulin (GLB) (A/G) ratio^b^	1–2.4	Ref					
	<1	0.425	1.831	0.176	1.53	0.826	2.831
	>2.4	−3.273	5.490	0.019^*^	0.038	0.002	0.586
Aspartate aminotransferase (AST) (U/L)^b^	5–34	Ref					
	<5	9.257	0.000	0.987	>999.999	<0.001	>999.999
	>34	−0.982	10.587	0.001^*^	0.375	0.207	0.677
Creatinine (μmol/L) ^b^	Male 64–104 Female 49–90	Ref					
	Male <64 Female <49	−0.308	1.188	0.276	0.735	0.422	1.279
	Male >104 Female >90	−1.058	7.968	0.005^*^	0.347	0.167	0.724

* *Indicates statistically significant results (P <0.05).*

a *The stepwise logistic regression analysis was used to explore the factors influencing COVID-19 prognosis.*

b *To explain the results more explicitly, these continuous variables were transformed into categorical variables according to their meanings and clinical reference ranges in the logistic regression analysis.*

*COVID-19, coronavirus disease-19*.

A poorer prognosis was associated with undergoing resuscitation (odds ratio [OR]: 0.067), developing critical illness during hospitalization (OR: 0.425), requiring consultation with outside specialists (OR: 0.077), basophil count > 0.06 ×10^9^ cells/L (OR: 0.396), lymphocyte count > 3.2 ×10^9^ cells/L (OR: 0.211), an A/G ratio > 2.4 (OR: 0.038), AST level > 34 U/L (OR: 0.375), male patients with creatinine level > 104 μmol/L, and female patients with a creatinine level > 90 μmol/L (OR: 0.347). A better prognosis was associated with initial symptoms of dry cough (OR: 6.17), the clinical manifestation of fatigue (OR: 1.96), and is diagnosed based on the highest level of evidence by means of biochemical and immunological tests (OR: 2.395).

## Discussion

In this study, 93.1% of the 1,765 patients with COVID-19 were cured, 4.8% improved, 0.2% were unhealed, and 1.8% died. Univariate analyses identified 63 significant factors for COVID-19 prognosis, while logistic regression analysis identified factors related to the severity of illness, symptoms and manifestations, diagnosis, and laboratory findings as factors independently associated with COVID-19 prognosis.

Patients with more severe diseases had a poorer prognosis. Undergoing resuscitation, consultation with outside specialists, and critical disease during hospitalization were all associated with more severe diseases. More critical patients were at a higher risk of death, which was consistent with the findings of a previous study, conducted in Wuhan, of 109 patients with COVID-19 who were serious enough to be admitted to the intensive care unit; all died owing to the rapid progress of the disease (29). Our finding was also indirectly supported by findings from a study from Detroit (23) and a study from Saudi Arabia (30) that emphasized admission to the intensive care unit would increase the incidence of death.

Patients who reported initial symptoms of dry cough and who had clinical manifestations of fatigue had a better prognosis. Our findings were consistent with those of other studies that found that though fatigue and dry cough are common symptoms and clinical manifestations, especially at the onset of illness (1, 31), among patients with a fatal disease, the most common symptom was dyspnea, followed by fatigue (29). Therefore, it was understandable that patients with a dry cough had a 6-fold better survival than those without this symptom and that those with the clinical manifestation of fatigue had double the survival of those without fatigue. However, some other studies have found the opposite association with survival. A study of 47 patients with COVID-19 in Xinyu, China (32) and a systematic review of 207 studies (33) found that fatigue was associated with a poorer prognosis. We were unable to find any studies that assessed dry cough as a prognostic indicator, and only a few studies have mentioned cough when describing the clinical characteristics of patients with COVID-19 (2, 34, 35).

Those diagnosed based on the highest level of evidence of biochemical and immunological tests were more than twice as likely to have a favorable outcome than patients with a diagnosis based on clinical findings. Considering that the admission period of patients in this study was from February 19, 2020, to March 19, 2020, the diagnosis and treatment were based on the *Diagnosis and Treatment Plan for COVID-19, Version 6.0* (36) *and Version 7.0* (37). The diagnostic criteria indicated that clinical diagnoses, along with radiological examinations (i.e., radiography and CT), should be used to identify suspected cases, while biochemical and immunological tests should be used for confirmation. Patients with confirmed COVID-19 were more likely to receive appropriate treatment, which could explain the better prognosis among patients diagnosed with COVID-19 based on the highest level of evidence of biochemical and immunological tests.

In terms of laboratory parameters, basophil count, lymphocyte count, A/G ratio, AST, and creatinine levels were associated with prognosis. Several previous studies have found that lymphocyte count is one of the most significant factors for COVID-19 prognosis and is a predictor of death (38). Most results of previous studies conducted in China and other countries found that lymphopenia was associated with a poorer prognosis (33, 35, 39–42), and one study found that a lymphocyte count <0.8 ×10^9^ cells/L was associated with an increased risk of severe COVID-19 (43). However, in our study, a lymphocyte count exceeding the upper limit of normal (>3.2 ×10^9^ cells/L) was associated with a poorer prognosis. Similarly, a study from Saudi Arabia found that the lymphocyte count of patients with moderate disease was higher than that of patients with mild disease (19), which indicated that the severity of the disease might be a confounding factor. Therefore, more evidence from larger samples in other countries or other regions of China is needed to explore the contradictory results of the lymphocyte count.

Concerning AST, a study from Libya (42) reached a similar conclusion as that of our study. The increased level of AST would increase the possibility of death.

Creatinine was another sensitive prognostic laboratory indicator. A higher level of creatinine was associated with a higher probability of death or severe disease. This finding was consistent with a study on 113 patients in China with fatal disease (7), a study on 1,207 patients in Libya (42), and a systematic review of 207 studies from multiple countries (33). Furthermore, to identify and care for patients with increasing creatinine levels, attention should be focused on male patients with creatinine levels >104 μmol/L and female patients with creatinine levels > 90 μmol/L.

Regarding other laboratory indicators with significance in univariate analyses in our study for which there was supporting evidence from national and international studies, WBC count (30, 33, 39, 42–45), percentage of lymphocytes (8), PLT count (8, 33, 42, 46–48), CRP level (8, 33, 43, 46, 48, 49), neutrophil count (33, 42, 43, 48), ALB level (33, 43, 49), blood UN (9, 33), and blood glucose level (45) were also associated with prognosis. However, some significant laboratory parameters, such as lactate dehydrogenase (7, 8, 33, 44, 45), D-dimer (7, 8, 10, 33, 43), and procalcitonin (5, 33, 44, 50), were not investigated in our study owing to a lack of data.

Furthermore, our study found that basophil count and A/G ratio were associated with COVID-19 prognosis, though few other studies have investigated these parameters. Extra attention should be paid to patients with a basophil count >0.06 ×10^9^ cells/L and A/G ratio > 2.4 to prevent disease progression. However, some controversial factors in previous studies were not significant to the prognosis in our study; these included sex and comorbidities (23, 24, 30).

There are some limitations to this study. First, it was a single-center study, which might have led to some biases. Further, the generalization of our findings is limited to some extent. Second, many patients were transferred from other hospitals and were not first diagnosed at MCH-Optical Valley; however, all laboratory indicators were tested on admission, implying that the laboratory findings of some patients were not their first test results—this might have introduced some biases. Third, data in this study were from February 19, 2020, to March 19, 2020, i.e., relatively old, though analyses were conducted on some inconsistent or previously unexplored indicators. Follow-up after discharge should be considered to compare the first wave with the following waves (e.g., the emergence of new variants) in future studies to provide further evidence for the prevention, diagnosis, and treatment of COVID-19. Fourth, some important factors (e.g., lactate dehydrogenase, D-dimer, and procalcitonin) investigated in other studies were not explored in this study owing to a lack of data. Fifth, this study had no control group, and the heterogeneity of the population might influence the generalization of the findings to other populations. Sixth, because some characteristics were self-reported, there existed some unreasonable classifications, such as viral pneumonia and tuberculosis in the reported initial symptoms. We did not make any corrections about these self-reported characteristics due to the lack of follow-up, which might bring some biases.

## Conclusions

The cure rate of 1,765 patients with COVID-19 in this study was 93.1%, and the mortality rate was 1.8%. To prevent the deterioration of the condition of the patient, clinicians should provide special care to patients developing critical illness during hospitalization, undergoing resuscitation, or needing consultation with outside specialists. In addition, patients with a basophil count >0.06 ×10^9^ cells/L, lymphocyte count > 3.2 ×10^9^ cells/L, an A/G ratio > 2.4, AST level > 34 U/L, and male sex with creatinine levels >104 μmol/L, or female sex with creatinine levels > 90 μmol/L were at a higher risk of having a poorer prognosis. Dry cough as an initial symptom, the clinical manifestation of fatigue, and being diagnosed with COVID-19 based on the highest level of evidence of biochemical and immunological tests were protective factors and was associated with a more favorable prognosis.

## Data Availability Statement

The raw data supporting the conclusions of this article will be made available by the authors, without undue reservation.

## Ethics Statement

The studies involving human participants were reviewed and approved by the 903rd Hospital of PLA Ethics Committee. Written informed consent from the participants' legal guardian/next of kin was not required to participate in this study in accordance with the national legislation and the institutional requirements.

## Author Contributions

All the authors contributed to the conception and design of the study. Material preparation, data collection, and analysis were performed by XiaL, LZ, TL, XibL, WY, and QC. The first draft of the manuscript was written by XiaL. All the authors have commented on previous versions of the manuscript and read and approved the final manuscript.

## Funding

This study was supported by the Zhejiang Provincial Natural Science Foundation of China (Grant No: LQ21H100001), the Soft Science Project of Shanghai Science and Technology Innovation Action Plan (Grant No: 21692191300), and the National Natural Science Foundation of China (Grant Nos: 82101870 and 72104140).

## Conflict of Interest

The authors declare that the research was conducted in the absence of any commercial or financial relationships that could be construed as a potential conflict of interest.

## Publisher's Note

All claims expressed in this article are solely those of the authors and do not necessarily represent those of their affiliated organizations, or those of the publisher, the editors and the reviewers. Any product that may be evaluated in this article, or claim that may be made by its manufacturer, is not guaranteed or endorsed by the publisher.

## Abbreviations

AST: Aspartate aminotransferase
COVID-19: Coronavirus disease
CRP: C-reactive protein
CT: Computed tomography
D-Bil: Direct bilirubin
DBP: Diastolic blood pressure
GGO: Ground-glass opacity
MCH: Maternal and Child Hospital of Hubei
MCHC: Mean corpuscular hemoglobin concentration
PLT, Platelet, RBC, Red blood cell, RDW, Red blood cell distribution width, SBP, Systolic blood pressure, TP, Total protein, UN, Urea nitrogen, WBC, White blood cell, WHO: World Health Organization.
