# Supplementary material for: Epidemiologic Characteristics of and Prognostic Factors for COVID-19 Among Hospitalized Patients: Updated Implications From Hubei Province, China
Source: Front Public Health. 2021 Oct 27;9:726491. doi: 10.3389/fpubh.2021.726491 (PMC8578829; doi:10.3389/fpubh.2021.726491)
Supplement: Supplementary file 1 [file Table_1.pdf]

Supplementary Table 1 Univariate analysis of the prognosis and different characteristics (continuous variables) of COVID patients.

Characteristic[a]	Mean[b]	Median (Interquartile ranges)[b]	Standard deviation[b]	Statistic[c]	*P*-value[c]
Age (years)	58.7	60.0 (50.0-68.0)	15.1	-0.114	<0.0001*
Length of stay (days)	13.3	12.0 (9.0-17.0)	6.1	0.013	0.579
Body temperature on admission (℃)	36.5	36.4 (36.2-36.7)	0.4	-0.007	0.784
Pulse rate on admission (count / min)	86.3	84.0 (78.0-95.0)	14.6	-0.043	0.070
Respiratory rate on admission (count / min)	19.4	20.0 (18.0-20.0)	3.5	-0.045	0.059
Systolic blood pressure (SBP) on admission (mmHg)	132.5	131.0 (120.0-142.0)	17.7	-0.016	0.490
Diastolic blood pressure (DBP) on admission (mmHg)	83.1	82.0 (75.0-90.0)	12.6	0.004	0.879
White blood cell (WBC) count ($\times 10^9$/L)	6.1	5.7 (4.7-6.8)	3.0	-0.054	0.024*
Percentage of lymphocytes (%)	28.5	28.6 (23.0-34.3)	9.0	0.108	<0.0001*
Percentage of eosinophils (%)	2.8	2.3 (1.4-3.5)	2.6	0.096	<0.0001*
Neutrophil count ($\times 10^9$/L)	3.9	3.5 (2.7-4.3)	2.5	-0.084	0.000*
Monocyte count ($\times 10^9$/L)	0.4	0.4 (0.3-0.5)	1.0	-0.003	0.913
Basophil count ($\times 10^9$/L)	0.0	0.0 (0.0-0.0)	0.1	0.057	0.016*
Hemoglobin (Hgb) level (g/L)	128.1	129.0 (119.0-139.0)	16.9	0.044	0.066
Mean corpuscular volume (MCV) (fL)	91.7	92.5 (89.5-95.0)	6.2	-0.001	0.980
Mean corpuscular hemoglobin concentration (MCHC) (g/L)	338.4	339.0 (333.0-345.0)	11.7	0.053	0.026*
Platelet (PLT) count ($\times 10^9$/L)	223.6	218.0 (179.0-257.0)	72.1	0.054	0.024*
Percentage of neutrophils (%)	61.5	61.3 (55.0-67.3)	10.0	-0.113	<0.0001*
Percentage of monocytes (%)	6.8	6.6 (5.6-7.7)	2.2	0.061	0.011*
Percentage of basophils (%)	0.4	0.4 (0.2-0.5)	0.8	0.112	<0.0001*
Lymphocyte count ($\times 10^9$ /L)	1.6	1.6 (1.3-2.0)	0.6	0.110	<0.0001*
Eosinophil count ($\times 10^9$ /L)	0.2	0.1 (0.1-0.2)	0.2	0.080	0.001*
Red blood cell (RBC) count ($\times 10^{12}$ /L)	4.1	4.1 (3.8-4.5)	0.6	0.022	0.346
Packed cell volume (PCV)	37.7	37.9 (35.1-40.7)	4.8	0.033	0.172
Mean corpuscular hemoglobin (MCH) (pg)	31.1	31.3 (30.3-32.4)	2.4	0.036	0.133
Red blood cell distribution width (RDW) (%)	13.2	13.0 (12.4-13.7)	1.5	-0.051	0.031*
Mean platelet volume (MPV) (fL)	9.8	9.7 (9.1-10.3)	1.0	-0.032	0.180
Blood glucose (mmol/L)	5.4	5.0 (4.4-5.6)	2.2	-0.109	<0.0001*
Albumin (ALB) (g/L)	38.5	38.8 (36.5-41.1)	4.2	0.118	<0.0001*
Albumin (ALB) / globulin (GLB) (A/G) ratio	1.2	1.2 (1.1-1.4)	0.5	0.100	<0.0001*
Direct bilirubin (D-BiL) (μmol/L)	4.7	4.1 (3.2-5.3)	3.8	-0.061	0.011*
Alanine aminotransferase (ALT) (U/L)	30.2	21.4 (13.6-32.8)	42.8	-0.024	0.306
Alkaline phosphatase (ALP) (IU/L)	74.2	71.0 (59.0-82.0)	31.7	-0.086	0.000*
Total bile acid (TBA) (μmol/L)	5.2	3.9 (2.4-6.0)	6.4	-0.043	0.071

Characteristic[a]	Mean[b]	Median (Interquartile ranges)[b]	Standard deviation[b]	Statistic[c]	*P*-value[c]
Sodium (mmol/L)	140.1	140.0 (139.0-141.0)	3.6	0.060	0.012[*]
Calcium (mmol/L)	2.2	2.2 (2.1-2.3)	0.2	0.035	0.138
Serum magnesium (mmol/L)	0.9	0.8 (0.8-0.9)	0.6	0.024	0.323
Urea nitrogen (UN) (mmol/L)	5.0	4.7 (3.9-5.5)	2.5	-0.111	<0.0001[*]
Uric acid (UA) (μmol/L)	308.1	302.0 (250.0-349.0)	88.6	-0.007	0.765
Total protein (TP) (g/L)	71.0	71.0 (67.4-74.4)	6.2	0.067	0.005[*]
Globulin (g/L)	32.4	31.9 (29.5-34.5)	4.9	-0.029	0.217
Total bilirubin (T-BiL) (μmol/L)	11.3	9.7 (7.5-12.7)	11.6	-0.036	0.128
Indirect bilirubin (I-BiL) (μmol/L)	6.6	5.7 (4.2-7.4)	7.6	-0.014	0.558
Aspartate aminotransferase (AST) (U/L)	21.2	16.7 (13.1-22.1)	25.7	-0.121	<0.0001[*]
γ -glutamyl transpeptidase (γ -GT) (U/L)	37.0	27.0 (18.0-40.0)	43.5	-0.025	0.291
Kalium (mmol/L)	4.3	4.1 (3.7-4.4)	1.5	0.009	0.697
Chlorine (mmol/L)	105.5	106.0 (104.0-107.0)	3.3	0.111	<0.0001[*]
Phosphorus (mmol/L)	1.2	1.16 (1.0-1.3)	0.2	0.034	0.151
Total carbon dioxide (TCO2) (mmol/L)	23.9	23.9 (22.5-24.9)	3.6	-0.009	0.696
Creatinine (μmol/L)	70.1	65.8 (56.6-75.6)	42.5	-0.054	0.024[*]
C-reactive protein (CRP) (mg/L)	12.0	1.7 (0.6-5.9)	29.0	-0.157	<0.0001[*]

[*] Indicates statistically significant results ($p<0.05$).

[a] All these continuous variables had a skewed distribution, which were tested by Kolmogorov-Smirnov test, with all $P < 0.05$.

[b] The mean, median (interquartile ranges), and standard deviation of these continuous variables were calculated by the descriptive statistics.

[c] The univariate analyses of these continuous variables were conducted by the Spearman rank correlation test.
