# Supplementary material for: Epidemiologic Characteristics of and Prognostic Factors for COVID-19 Among Hospitalized Patients: Updated Implications From Hubei Province, China
Source: Front Public Health. 2021 Oct 27;9:726491. doi: 10.3389/fpubh.2021.726491 (PMC8578829; doi:10.3389/fpubh.2021.726491)
Supplement: Supplementary file 2 [file Table_2.pdf]

Supplementary Table 2 Univariate analysis of the prognosis and different characteristics (categorical variables) of COVID patients.

Characteristics	Prognosis at discharge				Statistic[a]	P-value[a]
	Dead	Unhealed	Improved	Cured		
**Sex**					-1.887	0.059
Male	18	3	39	674		
Female	14	1	46	970		
**Marital status**					27.057	<0.0001*
Unmarried	0	0	1	66		
Married	19	4	77	1482		
Widowed	12	0	5	73		
Divorced	1	0	2	23		
**Occupation**					1488.791	<0.0001*
Worker	1	1	6	78		
Farmer	3	1	3	68		
Office worker	0	0	11	168		
Civil servant	0	0	2	42		
Professional and technological worker	0	0	1	47		
Self-employed person	0	0	2	42		
Freelancer	0	1	3	111		
Student	0	0	0	16		
Retiree	22	1	41	700		
Unemployed	3	0	10	184		
Others	3	0	6	188		
**Exposure history (contact with someone with confirmed or suspected COVID-19 during the 2 weeks preceding the onset of illness)**					39.330	<0.0001*
No	0	1	61	936		
Yes	1	0	16	460		
Unknown	31	3	8	248		
**Relationship between patients and people with COVID-19 to whom they were exposed**					9.449	0.051
Family members	0	0	12	318		
Colleagues	0	0	1	35		
Social interaction	0	0	2	48		

Characteristics	Prognosis at discharge				Statistic[a]	P-value[a]
	Dead	Unhealed	Improved	Cured		
Shared transportation	0	0	1	11		
Others	32	4	69	1232		
**Source of exposure**					24.964	0.001*
Eating together	0	0	7	118		
Staying in the same room	0	0	6	212		
Living in the same ward	0	0	1	16		
Sharing utensils with patients	0	0	0	5		
Contacting with patient secretions	0	0	0	4		
Treatment and care	0	1	58	769		
Visiting patient	0	0	0	5		
Others	32	3	13	515		
**Time period of exposure**					14.840	0.001*
Prolonged	0	0	10	335		
Brief	0	0	3	97		
Uncertain	32	4	72	1212		
**Possible location of exposure**					9.518	0.090
Home	0	0	12	295		
Workplace	0	0	1	39		
Dormitories	1	0	1	11		
Hospitals	0	0	1	30		
Indoor public places	0	0	4	36		
Others	31	4	66	1233		
**Admission route**					3.313	0.346
Emergency department	1	0	1	21		
Outpatient department	1	0	5	41		
Referral from other hospitals	3	0	7	109		
Others	27	4	72	1473		
**Illness condition on admission**					3.342	0.342
Without symptoms	0	0	1	9		
Unknown	0	0	1	2		
Clinically uncertain	1	0	0	19		
With symptoms	31	4	83	1614		
**Critical degree on admission**					170.592	<0.0001*
Dangerous	19	0	8	45		
Emergent	8	0	10	48		
Moderate	5	4	67	1551		

Characteristics	Prognosis at discharge				Statistic[a]	P-value[a]
	Dead	Unhealed	Improved	Cured		
**Undergoing surgery during hospitalization**					-3.646	0.000*
No	29	4	85	1639		
Yes	3	0	0	5		
**Undergoing resuscitation during hospitalization**					-16.606	<0.0001*
No	2	4	81	1620		
Yes	30	0	4	24		
**Developing critical illness during hospitalization**					-12.892	<0.0001*
No	0	4	60	1495		
Yes	32	0	25	149		
**Requiring consultation during hospitalization**					144.350	<0.0001*
No	10	4	76	1568		
In-hospital consultation	13	0	8	72		
Consultation with outside specialists	9	0	1	4		
**Being complex cases during hospitalization**					-9.047	<0.0001*
No	20	4	82	1625		
Yes	12	0	3	19		
**Reported initial symptoms**						
Fever	14	3	45	897	-0.730	0.465
Cough	13	1	42	731	-0.355	0.723
Expectoration	4	1	7	100	-1.703	0.089
Dry cough	0	0	2	157	2.939	0.003*
Stuffy nose and / or runny nose	1	0	0	17	0.171	0.864
Pant	5	0	9	148	-0.966	0.334
Shortness of breath	4	0	5	117	-0.169	0.866
Fatigue	6	2	18	511	2.213	0.027*
Chest distress and / or chest pain	3	0	16	218	-0.711	0.477
Dizziness and / or headache	0	1	1	26	-0.067	0.946
Abdominal pain and / or diarrhea and / or bloating	0	0	1	37	1.054	0.292
Sore throat	0	0	2	56	1.065	0.287
Dyspnea	3	0	5	23	-4.244	<0.0001*
Hemoptysis	0	0	0	2	0.382	0.703

Characteristics	Prognosis at discharge				Statistic[a]	P-value[a]
	Dead	Unhealed	Improved	Cured		
Palpitation	1	0	0	18	0.229	0.819
Muscle pain	0	1	1	43	0.642	0.521
Chest and / or back pain	0	0	1	6	-0.748	0.455
Nausea and / or vomit	1	1	0	19	-0.556	0.578
Poor appetite	2	0	2	45	-0.401	0.688
Chill	0	0	0	25	1.365	0.172
Disturbance of consciousness and apathy	8	0	0	2	-9.677	<0.0001*
Viral pneumonia	0	0	0	2	0.382	0.703
Tuberculosis	0	0	0	1	0.269	0.788
Unknown	4	0	1	13	-3.697	0.000*
**Current symptoms during hospitalization**						
Fever	16	3	48	992	-1.092	0.275
Cough	16	1	48	1019	-1.828	0.068
Chest distress	4	0	20	366	0.667	0.505
Dyspnea	6	0	7	64	-3.631	0.000*
**Clinical manifestations**						
Fever	1	1	27	703	4.136	<0.0001*
Cough	1	0	34	765	3.873	0.000*
Catarrh of the upper respiratory tract	1	0	6	189	1.950	0.051
Chest distress	1	0	25	450	1.504	0.133
Dyspnea	1	0	14	161	-0.848	0.397
Fatigue	1	0	28	629	3.237	0.001*
Diarrhea	0	0	4	70	0.542	0.588
**Previous medical history**					-3.539	0.000*
Without	12	4	54	1193		
With	20	0	31	451		
**Self-reported underlying diseases**						
Hypertension	12	0	35	481	-2.198	0.028*
Diabetes	5	1	18	202	-2.372	0.018*
Coronary heart disease	4	0	3	93	-0.116	0.908
Chronic bronchitis	1	0	2	38	-0.124	0.901
COPD	0	0	2	7	-1.773	0.076
Hyperlipoidemia	0	0	0	23	1.308	0.191
Asthma	0	0	1	13	-0.020	0.984
Atrial fibrillation	4	0	0	8	-3.870	0.000*
Bronchiectasis	0	0	0	8	0.768	0.443

Characteristics	Prognosis at discharge				Statistic[a]	P-value[a]
	Dead	Unhealed	Improved	Cured		
Alzheimer's disease	3	0	1	11	-3.181	0.002*
Parkinson's disease	1	0	0	5	-1.033	0.302
**Smoking habits**					32.083	<0.0001*
No	1	1	72	1313		
Yes	0	0	5	69		
Unknown	31	3	8	262		
**Treatment**						
Oxygen therapy measures	30	3	34	610	-4.161	<0.0001*
Antiviral therapy	22	3	70	1160	1.814	0.070
Mechanical Ventilation	16	0	0	14	-10.740	<0.0001*
ECMO	1	0	0	3	-1.535	0.125
Circulatory support	0	0	0	2	0.382	0.703
Renal failure and renal replacement therapy	0	0	0	2	0.382	0.703
Blood purification therapy	0	0	0	2	0.382	0.703
Immunotherapy with tocilizumab	8	0	0	12	-6.253	<0.0001*
Severe or critical child cases	1	0	1	7	-1.866	0.062
Pregnancy with severe or critical illness	0	0	0	1	0.269	0.788
**Ground-glass opacity tested by CT or chest radiography**					1.825	0.068
No	1	1	5	203		
Yes	18	3	80	1433		
**Location of ground-glass opacity**					5.983	0.649
Only right lung	0	0	8	127		
Only left lung	1	0	4	88		
Multiple points of both lungs	17	3	65	1141		
Right and upper left lung	0	0	1	30		
Right and lower left lung	0	0	1	31		
Left and upper right lung	0	0	0	1		
Left and middle right lung	0	0	1	9		
Left and lower right lung	0	0	0	8		
Unknown	14	1	5	209		
**Other observations obtained from imaging**						
Bronchial vascular bundle thickening	6	0	29	458	-0.192	0.848
Swollen lymph node	4	0	14	156	-1.890	0.059

Characteristics	Prognosis at discharge				Statistic[a]	P-value[a]
	Dead	Unhealed	Improved	Cured		
Pleural effusion	7	1	7	76	-3.831	0.000*
**Outpatient and emergency diagnosis**					15.167	0.010*
Confirmed COVID-19 case	20	3	55	1253		
Clinically diagnosed COVID-19 case	5	1	16	244		
Suspected COVID-19 case	5	0	14	121		
Infected by novel coronavirus	2	0	0	12		
Viral pneumonia	0	0	0	13		
Hypertension	0	0	0	1		
**Admission diagnosis**					20.195	0.003*
Confirmed COVID-19 case	19	3	58	1249		
Clinically diagnosed COVID-19 case	5	1	15	268		
Suspected COVID-19 case	6	0	12	107		
Infected by novel coronavirus	2	0	0	12		
Viral pneumonia	0	0	0	5		
Hypertension	0	0	0	2		
Chronic bronchitis	0	0	0	1		
**Discharge diagnosis**					17.773	0.001*
Confirmed COVID-19 case	21	3	70	1442		
Clinically diagnosed COVID-19 case	8	1	13	178		
Suspected COVID-19 case	2	0	2	10		
Infected by novel coronavirus	1	0	0	13		
Pneumonia	0	0	0	1		
**Subtype of COVID-19 at discharge**					183.009	<0.0001*
Mild	0	1	0	24		
Normal	2	2	33	891		
Severe	1	0	8	51		
Critical	16	0	1	9		
Suspected	1	0	1	5		
Clinical diagnosis	6	1	14	173		
Unknown	6	0	28	491		
**Consistency between discharge and outpatient diagnoses**					6.246	0.044*
Consistent	31	4	84	1611		
Uncertain	0	0	1	1		

Characteristics	Prognosis at discharge				Statistic[a]	P-value[a]
	Dead	Unhealed	Improved	Cured		
Unknown	1	0	0	32		
**Consistency between admission and discharge diagnoses**					6.194	0.045*
Consistent	32	4	84	1635		
Uncertain	0	0	1	1		
Unknown	0	0	0	8		
**Consistency between preoperative and postoperative diagnoses**					15.139	0.002*
Inconsistent	0	0	0	14		
Consistent	3	0	16	106		
Uncertain	0	0	0	2		
Unknown	29	4	69	1522		
**Consistency between clinical and pathological diagnoses**					2.474	0.480
Inconsistent	0	0	0	14		
Consistent	0	0	2	11		
Uncertain	0	0	0	1		
Unknown	32	4	83	1618		
**Consistency between radiological and pathological diagnoses**					1.280	0.734
Inconsistent	0	0	0	14		
Consistent	0	0	2	21		
Uncertain	0	0	0	2		
Unknown	32	4	83	1607		
**The highest evidence of diagnosis**					15.736	0.003*
Clinical diagnosis	14	2	24	374		
Radiography, CT, ultrasound, endoscopy	1	0	8	98		
Biochemical and immunological test results	15	2	29	882		
Cytological blood smear test	1	0	3	17		
Unknown	1	0	21	273		

* Indicates statistically significant results (p<0.05).

[a] The univariate analyses of these categorical variables were conducted by Wilcoxon rank test or Kruskal-Wallis H test, according to the type of these categorial variables.

The Wilcoxon rank test was used to test the relationship between prognosis and dichotomous variables. The Kruskal-Wallis H test was used to test the relationship between prognosis and multivariate variables.
